# Preparation and Performance Characterization of Exploding Foil Initiator Based on ODPA-ODA Polyimide Flyer

**DOI:** 10.3390/polym14214604

**Published:** 2022-10-30

**Authors:** Zhiqing Wu, Fan Lei, Zhiqiang Zhan, Jiangshan Luo, Gao Niu, Zhaoguo Li, Tao Yi, Shufan Chen, Bo Yang, Qiubo Fu, Zhiming Zhang

**Affiliations:** 1 Laser Fusion Research Center, China Academy of Engineering Physics, Mianyang 621900, China; 2 Institute of Chemical Materials, China Academy of Engineering Physics, Mianyang 621900, China

**Keywords:** exploding foil initiator, polyimide, flyer, plasma, morphology, chemical structure

## Abstract

The exploding foil initiator (EFI) system has been extensively used in ignition and detonation sequences and proved to be of high safety and reliability. Polyimide is considered the ideal flyer material for EFI due to its excellent performance, including thermal stability, outstanding mechanical properties, high radiation resistance, and excellent dielectric properties. In this study, we prepared the EFI based on a polyimide (ODPA-ODA) flyer, which is spin-coated and solidified on patterned copper film in situ. The electric explosion test shows that the prepared EFI has good working performance, and the 4000 V working voltage drove the flyer to reach a maximum velocity of 5096 m/s. The polyimide morphology and chemical structure after the electric explosion was observed by microscope, SEM, XPS, and FTIR, which showed that the polyimide flyer underwent thermal deformation and complex chemical reactions during an electric explosion. A large number of polyimide bonds broke to form new carbonyl compounds, and the opening of aromatic rings was accompanied by the formation of aliphatic hydrocarbon chains. The morphology and chemical structure analysis after the electric explosion test will lay a foundation for us to further understand the working principle and evolution process of polyimide (ODPA-ODA) flyer.

## 1. Introduction

Exploding foil initiator (EFI) system is a kind of in-line ignition and initiation device [1,2] which can directly initiate secondary explosives by the impact of a flyer at the desired velocity. Compared to other means of initiating high explosives, the EFI has been proven to be of high safety and reliability [3]. Generally, EFI is composed of an exploding foil, flyer plate, accelerating chamber, and explosive. Under the action of high pressure and heavy current, exploding foil generates high-temperature and high-pressure plasma to drive high-speed flyer to detonate explosives. So far, a range of materials such as polyimide [2,4,5], parylene C [6], polymethyl methacrylate [7], polyester [8], silicon [9], and PC/metal alloy [10] have been designed as flyer plate. Of the flyer materials mentioned above, polyimide is the most attractive engineering polymer [11], which exhibits thermal stability, outstanding mechanical properties, high radiation resistance, and excellent dielectric properties. Due to its excellent performance, polyimide has been widely used in aerospace [12], microelectronics [13], liquid crystal [14], separation membranes [15], and other fields. PMDA-ODA polyimide is commonly used for the flyer in EFI, probably because of its excellent engineering performance and commercial availability. For example, Ichihara [4] recently evaluated the energy conversion efficiency of an EFI that accelerates a flyer of polyimide film. Borman and Dowding [5] demonstrated the use of numerical simulation to predict the mass of plasma formed and subsequent mass ejected from an EFI with polyimide flyer during various initial circuit conditions. Sanchez [16] and collaborators reported the in situ investigation of EFI during flight with synchrotron sources, which further improves our understanding of the behavior of polyimide flyers. Very recently, we have evaluated the effect of material properties on the performance of the EFI and revealed that thermal curing temperature during the final imidization process could influence the property of the polyimide material and flight ability of the flyer [17].

Traditionally, most of the flyer plates and the accelerating chamber used in EFI are manually pasted and aligned [18]. The assembly process is complicated, making it difficult to achieve scale production. Furthermore, the assembled device has poor stability, and high ignition energy is required. Therefore, improving production efficiency and reducing ignition energy have become major problems to be solved urgently for EFI. With the rapid development of microelectromechanical system (MEMS) technology, in situ integration of EFI has been receiving increasing attention in recent years due to the high processing accuracy and preparation efficiency [19]. For instance, Desai [20] proposed a bottom-to-top in situ preparation of McEFI using non-silicon MEMS technology. By means of deposition coating, photolithography, and imaging techniques, the explosive bridge foil, flyer plate, and accelerating chamber are deposited on the substrate successively. Zhu [21] and collaborators developed an electro-explosively actuated mini-flyer launcher capable of ejecting a plastic mini-flyer. With the MEMS methods, the process can be mass-produced and takes only four steps, lowering manufacturing costs.

Although significant advances have been made in the field of in situ integration of EFI, few studies have focused on preparing EFI based on polyimide flyer, as well as the morphologic and chemical structure changes of polyimide flyer after the electric explosion test. Herein, we report the preparation of EFI based on ODPA-ODA polyimide film, which is spin-coated and solidified on patterned copper film. The electrical explosion test shows that the flyer of polyimide film prepared in situ exhibit excellent performance. Several accessible investigation tools, such as scanning electron microscopy (SEM), energy-dispersive X-ray spectroscopy (EDX), X-ray photoelectron spectroscopy (XPS), and Fourier transform infrared spectroscopy (FTIR), were used to evaluate the modifications of polyimide film after the electric explosion test. This study will help us further understand the morphology and chemical structure changes of ODPA-ODA polyimide flyers during electrical explosions and provide more information for designing and preparing new and more efficient flyers in the future.

## 2. Materials and Methods

### 2.1. Materials

4,4′-Oxydiphthalic anhydride (ODPA) was purchased from J&K Scientific Ltd.; Oxydianiline (ODA) and N-Methyl pyrrolidone (anhydrous, 99.5%) were obtained from Aladdin (Shanghai, China). All the chemicals were used as received.

### 2.2. Fabrication of EFI Based on ODPA-ODA Polyimide Flyer

**Figure 1:**
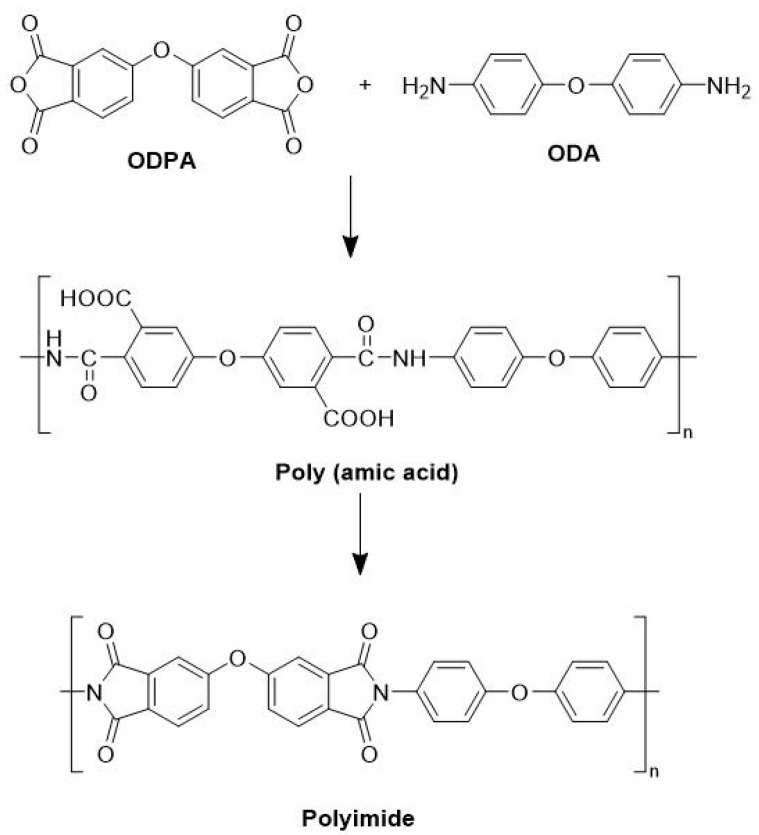
The synthesis procedure of ODPA-ODA polyimide.

**Figure 2:**
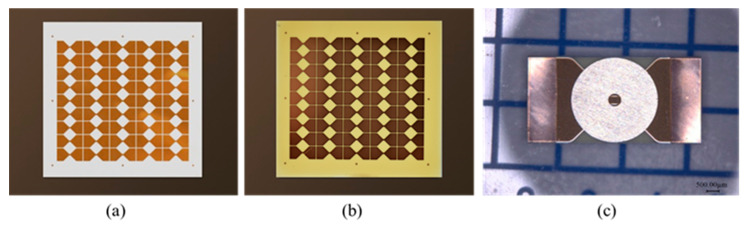
(**a**) Patterned copper film on the ceramic substrate, (**b**) ODPA-ODA polyimide film on the patterned copper film, (**c**) EFI devices with nickel barrel.

The polyamic acid is formed from the polycondensation reaction of 4,4,-Oxydiphthalic anhydride (ODPA) and oxydianiline (ODA) at room temperature [22], as shown in Figure 1. In a general way, we first coated the ceramic substrate with a copper film of 4 μm based on the magnetron sputtering method. Polyimide film was made by casting the polyimide precursor solution of ODPA-ODA polyamic acid on the patterned copper film after photoetching (Figure 2a) and then thermal imidization to form a solid film (Figure 2b) [15]. In detail, the polyamic acid solution was deposited uniformly on the copper film using a spin coating process by using a spin coater set at 1000 rounds per min for 25 s. The imidization process was performed in a vacuum oven for heating at a rate of 2 °C/min. During the curing process, the sample received an initial thermal treatment at 100 °C for 40 min, followed by a dehydration treatment at 200 °C for 60 min. The temperature was raised to 300 °C and maintained for another 60 min to complete the imination reaction [23]. Finally, the sample was then cooled under a vacuum to provide the 30 μm thick polyimide film. After that, the sample was cut to the appropriate size by a scribing machine, and nickel barrels were then adhered to the polyimide film surface to afford the EFI devices, as shown in Figure 2c.

### 2.3. Characterization

The nanomechanical properties of polyimide film are measured with a Nano-indenter (G200, Agilent, Santa Clara, CA, USA). The maximal penetration depth, strain rate, and harmonic displacement were 100 nm, 0.05 s^−1,^ and 1 nm, respectively. The microstructures of the polyimide film were investigated by a scanning electron microscope (SEM, Hitachi, SU8000, Tokyo, Japan). The chemical structures of the polyimide film were characterized by XPS (Escalab 250Xi, Thermo Fisher, Waltham, MA, USA), the elemental ratios were determined from the integral intensities of carbon 1s (C 1s), nitrogen 1s (N 1s), and oxygen 1s (O 1s) peaks. Fourier Transform infrared spectroscopy (FTIR) analysis was recorded by Nicolet iN10 spectrometer (Thermo Fisher, Waltham, MA, USA).

### 2.4. Electric Explosion Experiment

**Figure 3:**
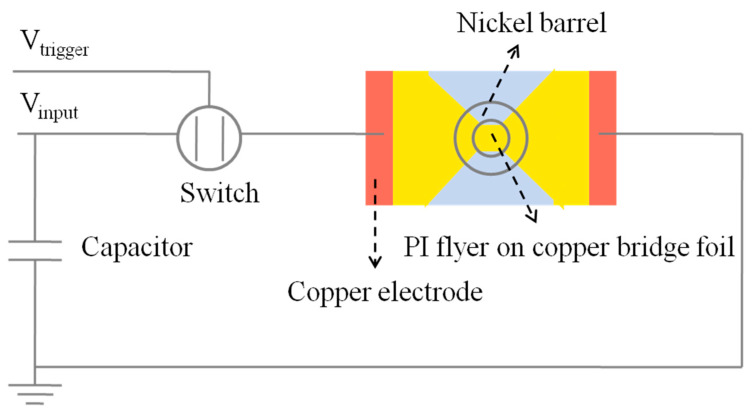
Schematic diagram of the electric explosion device.

To study the electrical explosion performance of the in situ prepared polyimide flyer, a photo doppler velocity (PDV) measurement system, based on the doppler frequency shift effect, is used to measure the velocity of the polyimide flyer [4,17]. The explosion current and voltage of the exploding foil were measured by a homemade device, whose schematic diagram is shown in Figure 3. The device consisted of the high-voltage pulse power source, the EFI device, and the signal acquisition system. The high-voltage pulse power source included the high-voltage power supply, the capacitor, and the trigger switch. The value of the capacitor was 0.31 μF. The working voltage was chosen to be 4000 V.

## 3. Results and Discussion

### 3.1. The Properties of Polyimide Film

First, we tested the mechanical and electrical properties of the polyimide film to prove that the flyer we prepared met the mechanical strength and insulation requirements of the EFI devices. Nano-indentation test showed that the modulus and hardness of polyimide film were 4.42 Gpa and 0.37 Gpa, as shown in Table 1. The dielectric constant of the film is 3.29. In addition, the voltage breakdown field strength and leakage current densities are 3.9 × 10^6^ v/m and 1.2 × 10^−4^ A/cm^2^, respectively. The results show that the polyimide film has good mechanical strength and dielectric properties [15].

### 3.2. The Performance of the EFI

**Figure 4:**
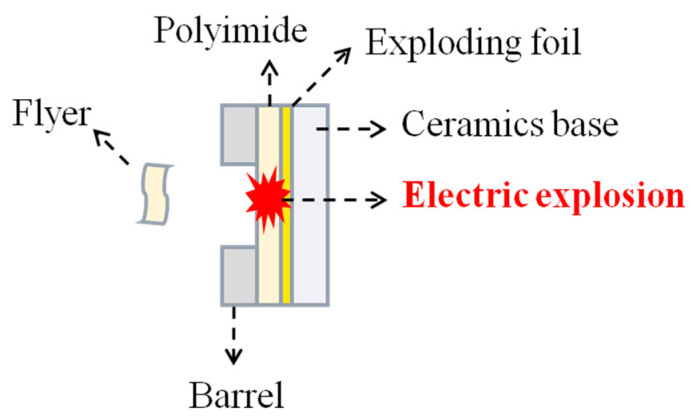
Diagrammatic sketch of the electrical explosion.

**Figure 5:**
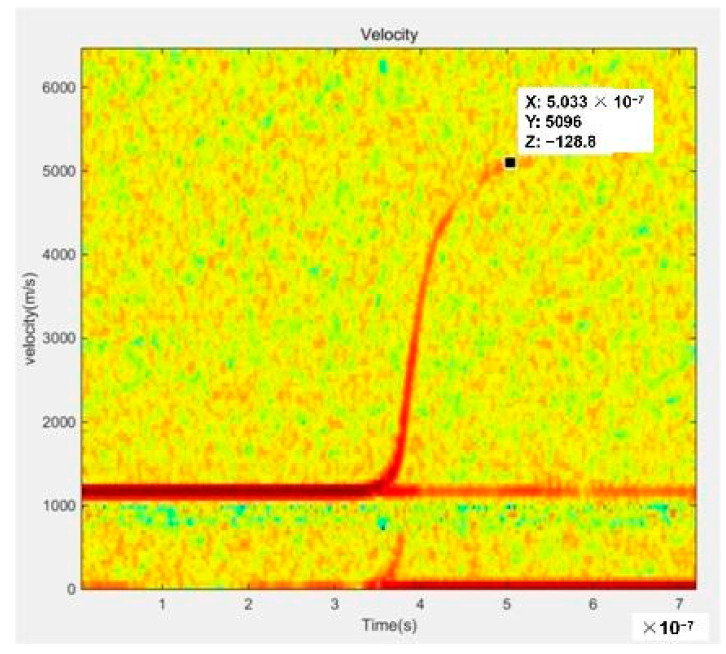
PDV spectrogram of the flyer with a working voltage of 4000 V.

We subsequently carried out an electrical explosion test to verify the working performance of the above-prepared EFI. As illustrated in Figure 4, under the action of a high pulse current, the explosive metal foil forms a high-temperature and high-pressure plasma, thus promoting the polyimide film escape as a flyer from the nickel barrel. Figure 5 shows the typical PDV spectrogram of the polyimide flyer at the working voltage of 4000 V. Note that the speed curve is smooth, which indicates that the flight ability of the flyer is very stable. Additionally, the 4000 V working voltage drove the flyer to a maximum velocity of 5096 m/s, demonstrating excellent electrical explosive performance. The electrical explosion test result of this unoptimized EFI device is comparable with the PMDA-ODA polyimide flyer reported previously [17].

### 3.3. The Morphology of Flyer Plate after the Electric Explosion Test

**Figure 6:**
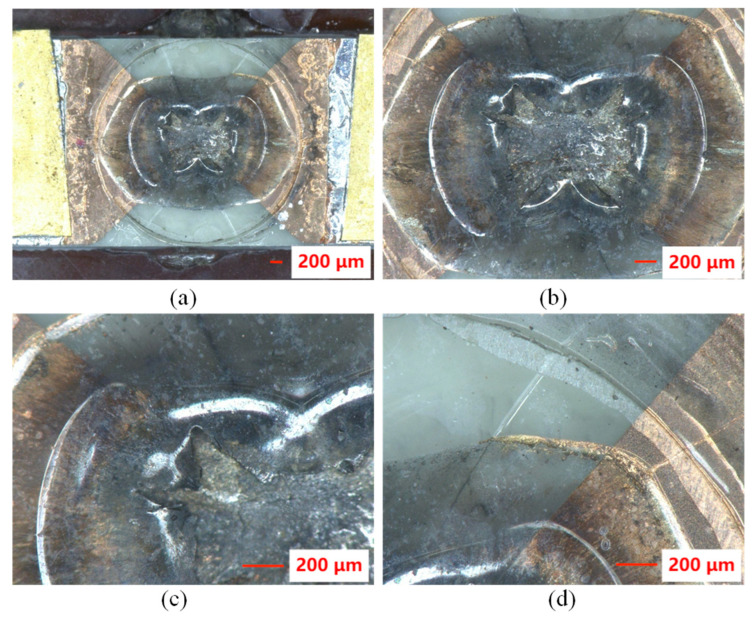
(**a**) Microscopic images of the flyer plate after the electric explosion test. (**b**–**d**) Enlarged view of the polyimide film.

The morphology characteristics of the flyer plate after the electric explosion test were analyzed, as shown in Figure 6. Microscopic images show that the polyimide film has a crater-like appearance after the electrical explosion experiment. In the explosion bridge area, the polyimide film is deformed and detached from the metal foil and ceramic substrate. There are two circular marks from the center of the explosion. This morphology may be mainly due to the expansion of polyimide film into a bubble shape by the impact of copper plasma during the electric explosion, and finally, the film shear in the central region to form a polyimide flyer [3]. A small amount of copper and uneven black material can be seen between the ceramic sheet and the polyimide film. During the electrical explosion, the copper film of the bridge area completes the transformation of solid, liquid, gas, and plasma in a very short time [24]. As the explosion progresses, the temperature decreases, and part of the copper is redeposited on the polyimide film. Some of the copper might be oxidized to copper oxide during the electric explosion, which is deposited on the polyimide film.

**Figure 7:**
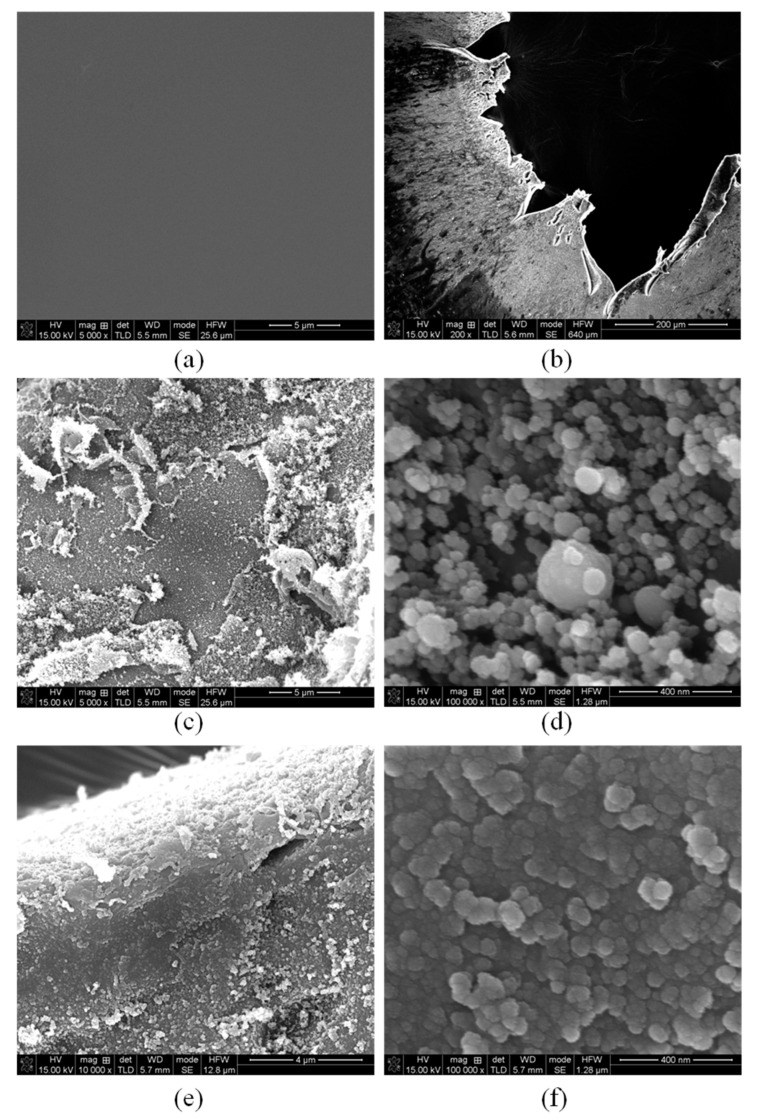
(**a**) SEM of the polyimide film flyer as prepared. (**b**–**f**) flyer plate after the electric explosion test. (**c**) and (**d**) display the morphology of the film surface attached to the ceramic substrate, (**e**,**f**) display the morphology of the film surface close to the barrel direction.

The surface morphology was further analyzed by SEM. First, the morphology of the polyimide film flyer as prepared displays a smooth surface, as shown in Figure 7a. Figure 7b shows the crater rim morphology of the polyimide film after the electric explosion test, which is exemplified in Figure 6. For the deformation region close to the crater rim, the film surface characteristics attached to the ceramic substrate and the surface close to the barrel direction were exhibited in Figure 7c–f, respectively. Both surfaces changed substantially, which showed clear circular colloidal morphology of tens of nanometers after the electric explosion test. In this work, a polyimide film is prepared by spin-coating based on the solution method. Under the impact of high temperature and high-pressure plasma, the thin film is heated and expanded, resulting in a loose colloidal shape on the surface. By comparison, the surface structure of the polyimide film close to the copper film is more loose than that close to the barrel. This is probably because the surface close to the copper film is impacted more directly and violently by the plasma.

### 3.4. Characterization of Chemical Composition of Polyimide Film Flyer after the Electric Explosion

To characterize the changes in the chemical composition of polyimide film flyer after the electric explosion, the surface was analyzed by Energy-dispersive X-ray spectroscopy (EDX). EDX was used to assess the compositional structure of the polyimide film surface before and after the electric explosion. After the electric explosion, we can see from EDX scanning that the surface of polyimide mainly contains four elements, including carbon, nitrogen, oxygen, and copper. The presence of copper is due to the plasma deposition of copper on the polyimide film during the electric explosion. A comparative view of the main constitutive elements of the polyimide film is depicted in Table 2 in terms of atomic concentration (%). Compared with pristine polyimide film, the relative content of carbon, nitrogen, and oxygen elements changed after the electric explosion test. The results indicate several changes in the chemistry of the PI films.

**Figure 8:**
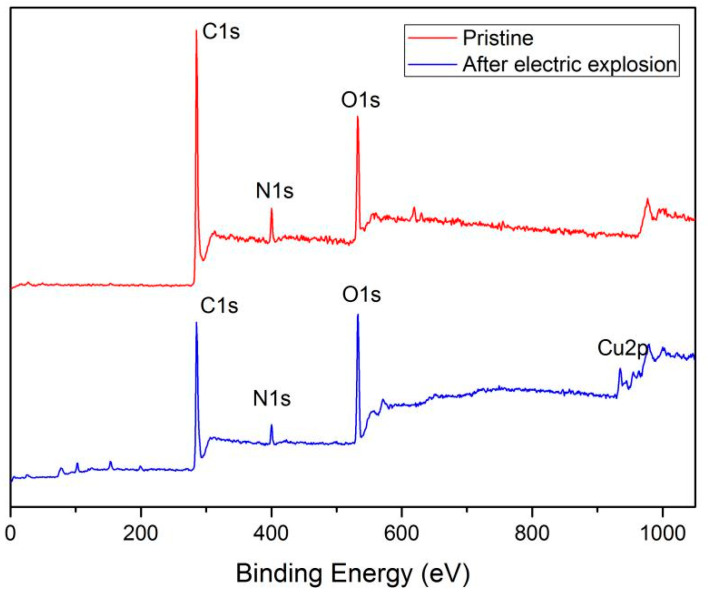
XPS spectra of pristine polyimide film and the polyimide film after the electric explosion.

Furthermore, the XPS was introduced to investigate the chemical modifications on the surface of the polyimide film flyer after the electric explosion test. Figure 8 shows the XPS spectra of the pristine polyimide film and the polyimide film after the electric explosion test. In addition to the carbon, nitrogen, and oxygen contained in polyimide, the sample of polyimide film after the electric explosion also contains a small amount of copper.

We focused on the variation of carbon, nitrogen, and oxygen elements contained in the polyimide molecules. According to the XPS spectra, the atomic concentration of C 1s, N 1s, and O 1s of the pristine film and sample after the electric explosion test are shown in Table 3. For the sample after the electric explosion test, we tested three points (P1, P2, P3) on the film surface of the deformation region close to the crater rim. We can see that the relative amounts of carbon and nitrogen drop dramatically after the electric explosion test. The content of elements in the three points showed the same trend. Conversely, in the three points tested, the relative amounts of oxygen increased from 17.63% to a maximum of 30.36%.

**Figure 9:**
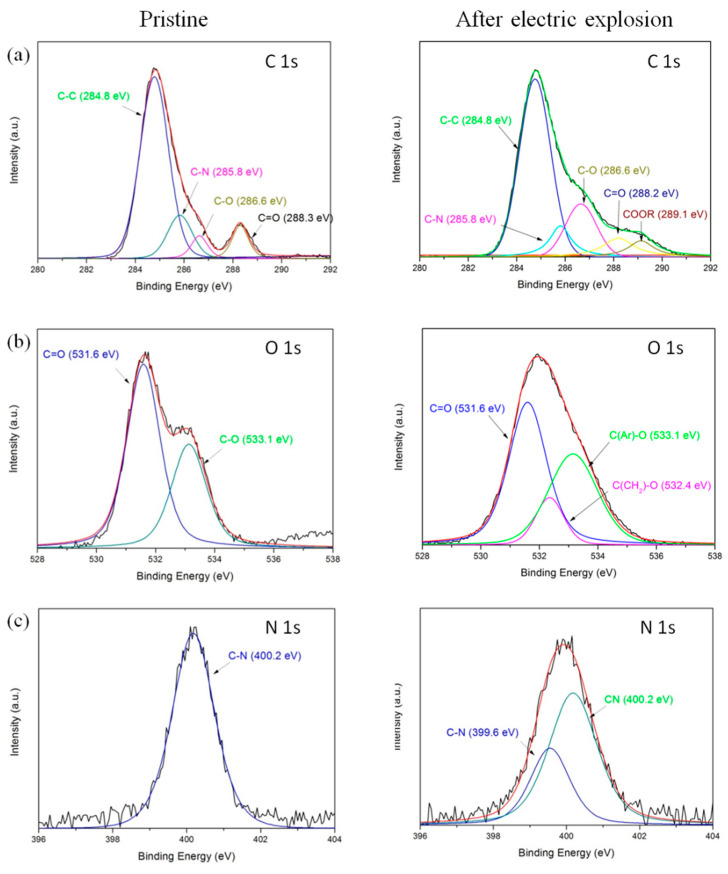
Curve-fitted XPS spectra of (**a**) C1s, (**b**) O1s and (**c**) N1s for pristine polyimide film and sample after the electric explosion test.

The XPS spectra of C 1s obtained on the surface of the pristine polyimide film are presented in Figure 9a. The prominent peak located at 284.8 eV corresponds to the carbon atoms of two benzene rings of the ODA structure. The peak at 285.8 eV is related to the C–N bonds. The other two peaks at 286.6 eV and 288.3 eV are attributed to ether group C–O in the ODA unit and carbonyl group C=O in the imide systems of the ODPA unit, respectively [25,26,27,28]. After the electric explosion test, the relative intensities of these peaks have changed significantly, and the detailed relative component ratios of different bonds are shown in Table 4. The relative intensities of C–C and C–N bonds decrease, and meanwhile, the relative intensities of C–O and C=O bonds increase. Importantly, a new chemical bond was detected at 289.1 eV, which can be assigned to a carboxyl bond [28]. These results suggest that new oxygen-containing functional groups have been introduced into the polyimide film surface with the break of the C–C and C–N bonds during the electric explosion. During the plasma treatment, part of the molecular chain might be changed to carboxylic acid via a ring-opening process.

Figure 9b shows the O1s spectra of polyimide film. Two deconvoluted signals are detected at 531.6 eV and 533.1 eV associated with carbonyl group C=O and ether group (Ar–O), respectively. The ratio of C=O decreases significantly after the electric explosion test. Meanwhile, a new chemical bond was detected at 532.4 eV, which can be assigned to the ether group (CH_2_–O) [27]. There might be severe chemical bond breakage of the benzene ring during plasma treatment, which facilitates the generation of aliphatic C–C bonds and the corresponding aliphatic ether group.

Figure 9c shows the N1s spectra of polyimide film, where only one symmetric peak at 400.2 eV (C–N) is detected for the pristine sample. After the electric explosion test, a new peak at 399.6 eV is detected, assigned to the C≡N bond. The formation of C≡N bonds has also been observed when polyimide is irradiated by a high-energy electron beam and coupling irradiation treatment [27].

The results indicate that complex chemical reactions occur on the polyimide film surface after the electric explosion test. The main damage mechanism is the breakage of polyimide bonds and the formation of some new carbonyl compounds. At the same time, under the condition of high temperature and high-pressure electric explosion, the ring-opening reaction of the aromatic ring takes place, accompanied by the formation of aliphatic hydrocarbon chains and corresponding oxide.

**Figure 10:**
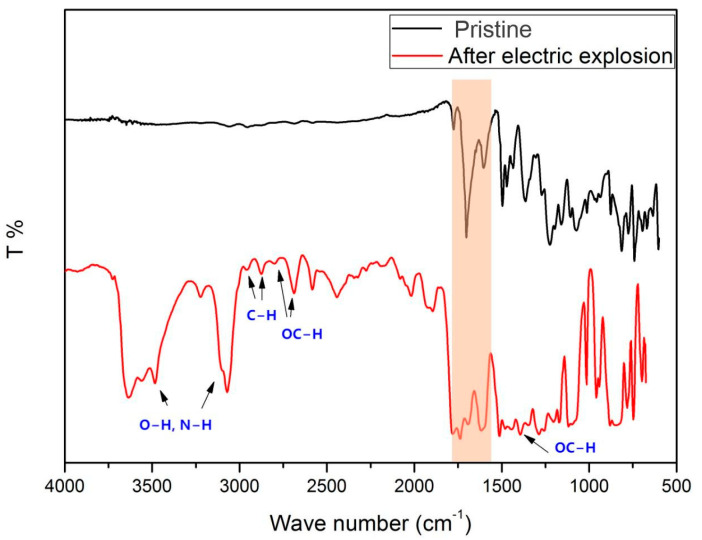
FTIR spectra for pristine polyimide film and sample after the electric explosion test.

Then, the surface molecular structures of the pristine polyimide film and after the electric explosion test were characterized by FTIR. Typically, the analysis and the locations of the main functional groups are shown in Figure 10. The peaks at 1775 and 1709 cm^−1^ are attributed to the asymmetrical and symmetrical stretching vibrations of the carbonyl groups (C=O) in the imide. The peaks at 1380 and 1240 cm^−1^ are the vibration peak of the C–N bond. The peaks at 1496 and 1600 cm^−1^ are attributed to the stretching vibrations of the carbon backbone (C=C) of the benzene ring [21]. The comparison of the FTIR spectra before and after the electric explosion test indicates that the position of infrared absorption peaks did not change significantly while a large number of new absorption peaks appeared. New absorption peaks appeared around 1700 cm^−1^, indicating the emergence of new carbonyl compounds due to the breakage of the polyimide bond. In addition, the peaks at 1391, 2689, and 2800 cm^−1^ are commonly considered as Fermi Resonance and C–H distortion vibration of the aldehyde group. The presence of absorption peaks at 2960 and 2876 cm^−1^ confirms the presence of aliphatic hydrocarbons, which is consistent with the XPS analysis results. The new absorption peaks in the 3100–3700 cm^−1^ range were characteristic absorption bands of amino, carboxyl and hydroxyl groups, demonstrating the massive polyimide bond breaking on the film surface.

## 4. Conclusions

In this study, we prepared the EFI based on in situ preparation of ODPA-ODA polyimide film as the flyer. The polyimide film prepared by in situ spin coating shows good mechanical and dielectric properties, which is expected to be an excellent flyer material. The electric explosion test indicates that the prepared EFI has good working performance. The microscope and scanning electron microscope show that the flyer underwent thermal deformation during the electric explosion. XPS and FTIR analysis demonstrate the polyimide film underwent complex chemical reactions under the high temperature and high pressure of the electric explosion. A large number of polyimide bonds broke to form new carbonyl compounds, and the opening of aromatic rings was accompanied by the formation of aliphatic hydrocarbon chains. The morphology and chemical structure analysis after the electric explosion test will lay a foundation for us to further understand the working principle and change process of the electric explosion. At the same time, this work provides more information for us to design new efficient flyer materials and structures in the future.

## Figures and Tables

**Table 1:** Mechanical and electrical properties of the polyimide film.

Property	Test Data
Modulus	4.42 Gpa
Hardness	0.37 Gpa
Dielectric constant	3.29 (1 MHz, 18 °C)
Voltage breakdown field strength	3.9 × 10^6^ V/m
Leakage current density	1.2 × 10^−4^ A/cm^2^

**Table 2:** The atomic ratio of C, O, and N in pristine polyimide film and the sample after the electric explosion test.

Sample	Atomic Concentration (%)			
	C	O	N	Cu
Pristine polyimide film	72.85	20.87	6.29	-
Polyimide film after the electric explosion test	70.51	22.5	6.91	0.08

**Table 3:** The atomic ratio of C, O, and N in pristine polyimide and the sample after the electric explosion test.

Sample		Atomic Concentration (%)		
		C 1s	O 1s	N 1s
Pristine polyimide film		75.41	17.63	6.96
Polyimide film after the electric explosion	P1	70.52	24.06	5.42
	P2	64.58	30.36	5.07
	P3	70.4	24.67	4.93

**Table 4:** The component ratios of pristine and the sample after the electric explosion test.

Peaks	Bonds	E_bind_ (eV)	Proportion (%)	
			Pristine	after The Electric Explosion
C1s	C–C	284.8	70.93	57.49
	C–N	285.8	14.89	10.2
	C–O	286.6	5.77	16.88
	C=O	288.3	8.41	9.1
	COOR	289.1	0	6.33
O1s	C=O	531.6	62.89	50.49
	CH_2_–O	532.4	0	11.41
	Ar–O	533.1	37.11	38.1
N1s	C–N	400.2	100	63.1
	C≡N	399.6	0	36.9

## Data Availability

The data presented in this study are available on request from the corresponding author.
